# Sacrum osteosarcoma after pelvic radiation for uterine cervical cancer: highlighted issues

**DOI:** 10.1590/S1516-31802012000500013

**Published:** 2012-11-13

**Authors:** Ramon Andrade De Mello

**Affiliations:** I MD. Assistant Professor, Department of Medicine, School of Medicine, University of Porto, and Department of Medical Oncology, Portuguese Oncology Institute, Porto, Portugal.

## INTRODUCTION

Osteosarcoma is a rare but serious sequel of radiotherapy.^1^ The incidence of sarcomas post-irradiation therapy comprises about 0.1% of all cancer cases and the sarcomas usually appear 10 to 14.3 years post-treatment.^1^^,^^2^ Women are expected to suffer these sequels because they are more frequently subjected to radiotherapy for breast and gynecological cancers and they have better long-term survival.^2^ Here, the difficulties of clinical management will be briefly addressed and also some issues in the literature on this field will be discussed.

### Squamous cell carcinoma

Squamous cell carcinoma (SCC) accounts for about 80% of all cervical cancers.^3^ Over the last few decades, primary external radiotherapy has usually been the first neoadjuvant treatment choice for cervical SCC stages IB2 and IIA2.^4^ In 1999, it was demonstrated that radiotherapy associated with cisplatin, 40 mg/m^2^ of body surface area once a week for up to six cycles, with a maximum weekly dose of 70 mg/m^2^ (chemoradiotherapy), improved the outcome for these patients.^5^^,^^6^

### Osteosarcoma

Osteosarcoma is a rare and aggressive disease that typically presents with symptoms of pain or swelling.^7^ Histologically, it consists of an aggressive spindle cell neoplasm that produces osteoid matrix. In older adults, it usually occurs as a result of sarcomatous transformation of Paget’s disease of bone.^7^

In 1922, Beck were the first to describe osteosarcoma following radiotherapy, in a series of three cases.^8^ More recently, it has also been demonstrated that angiogenesis may play a leading role in its pathogenic molecular mechanisms, through overexpression of vascular endothelial growth factor in high-grade osteosarcomas.^9^

Although the overall annual incidence of osteosarcoma comprises about one case per 100,000 individuals,^7^ it has also been demonstrated that osteosarcoma could be related to pelvic radiation therapy.^1^^,^^10^ Patients might remain in a state of latency for 11 years post-radiotherapy, until the osteosarcoma diagnosis is made. In a recent report,^11^ osteosarcoma was considered to have been caused by radiation treatment for uterine cervical cancer (UCC), as a late sequel in two patients: one with UCC stage IIB and the other with stage IIIB.

Surgery is the expected approach for osteosarcoma control, either for primary or for metastatic disease, when it is resectable.^7^ In this situation, palliative chemotherapy is important to keep the patient in a state of stable disease and good general condition.

In 1993, a phase III study showed that MAID (mesna, doxorubicin, ifosfamide and dacarbazine) chemotherapy was effective for treating adult high-grade osteosarcoma.^12^ Nevertheless, a phase II study, in 1998, showed lower response and survival rates for this chemotherapeutic regimen among patients with osteosarcoma.^13^ It was therefore suggested that a combination of doxorubicin and cisplatin should remain the first-line regimen for patients with osteosarcoma. Furthermore, it was found that the lungs were the most frequent metastasis site due to osteosarcoma.^14^

### CONCLUSION

Malignant bone disease is a well-known but quite rare sequel post-radiotherapy, as previous described.^15^ This particular situation is of interest not only because of its rarity but also because of the way in which osteosarcoma is sometimes diagnosed and especially because of the length of time over which patients may remain stable subsequent to MAID chemotherapy, with good performance status, and without disease progression, even without any surgical approach towards the bone sarcoma.
